# Immune checkpoint inhibitor-induced subacute cutaneous lupus erythematosus: a case report and review of the literature

**DOI:** 10.3389/fmed.2024.1334718

**Published:** 2024-02-01

**Authors:** Adam Khorasanchi, Abraham M. Korman, Ashish Manne, Alexa Meara

**Affiliations:** ^1^Division of Medical Oncology, The Ohio State University Comprehensive Cancer Center, Columbus, OH, United States; ^2^Department of Dermatology, The Ohio State University, Columbus, OH, United States; ^3^Division of Rheumatology and Immunology, The Ohio State University, Columbus, OH, United States

**Keywords:** cutaneous lupus erythematosus, esophageal cancer, immune checkpoint inhibitor, case report, literature review

## Abstract

Immune checkpoint inhibitor (ICI) use has been associated with numerous autoimmune side effects, known as immune related adverse events (irAEs). Cutaneous irAEs are common and affect up to 50% of patients treated with ICIs. There have been an increasing number of cases reported in the literature regarding ICI-induced subacute cutaneous lupus erythematosus (SCLE). ICI-induced SCLE is important to recognize as it can result in a delayed and/or prolonged skin reaction despite treatment discontinuation. We describe a patient with gastro-esophageal adenocarcinoma who developed SCLE following one cycle of nivolumab treatment. A 75-year-old man presented to our clinic with a new photo-distributed rash composed of oval scaly pink papules and plaques involving his chest and arms. Despite treatment with topical corticosteroids, he presented to the emergency department 1 week later with worsening rash. Skin biopsy showed vacuolar interface pattern, along with superficial perivascular lymphocytic infiltrate, consistent with a drug eruption. The clinicopathological presentation was consistent with ICI-induced SCLE. Nivolumab treatment was discontinued due to the severity of the rash. The rash remitted with systemic corticosteroids, high potency topical steroids, and hydroxychloroquine. Unfortunately, the patient developed intraperitoneal metastatic disease, and was enrolled in hospice care. In this paper, we highlight the importance of early identification and treatment of this irAE. A review of the literature, including a discussion on the management of ICI-induced SCLE is also provided.

## Introduction

Immune checkpoint inhibitors (ICIs) have revolutionized the care of patients with advanced solid tumors since their initial FDA approval for metastatic melanoma (1). ICIs exert their antitumor effects via immune checkpoint blockade which can overcome tumor mediated T cell inhibition and result in a durable treatment response, allowing the body to attack the tumor itself. However, ICI exposure can also result in immune hyperactivation, and the development of autoimmune sequela called immune related adverse events (irAEs) (2, 3). Cutaneous irAEs are the most frequently reported irAEs, affecting 30%–50% of patients treated with ICIs, and have been associated with improved anti-tumor efficacy (4–6). There have been an increasing number of cases reported in the literature regarding ICI-induced subacute cutaneous lupus erythematosus (SCLE) (7–10). While drug-induced SCLE typically resolves following discontinuation of the offending medication, ICI-induced SCLE is a distinct entity, which can result in a delayed and/or prolonged skin reaction despite treatment discontinuation (11, 12). In this paper, we report a case of ICI-induced SCLE in a patient with gastro-esophageal adenocarcinoma. A review of the literature, including a discussion on the management of ICI-induced SCLE is also provided.

## Case description

**Figure 1:**
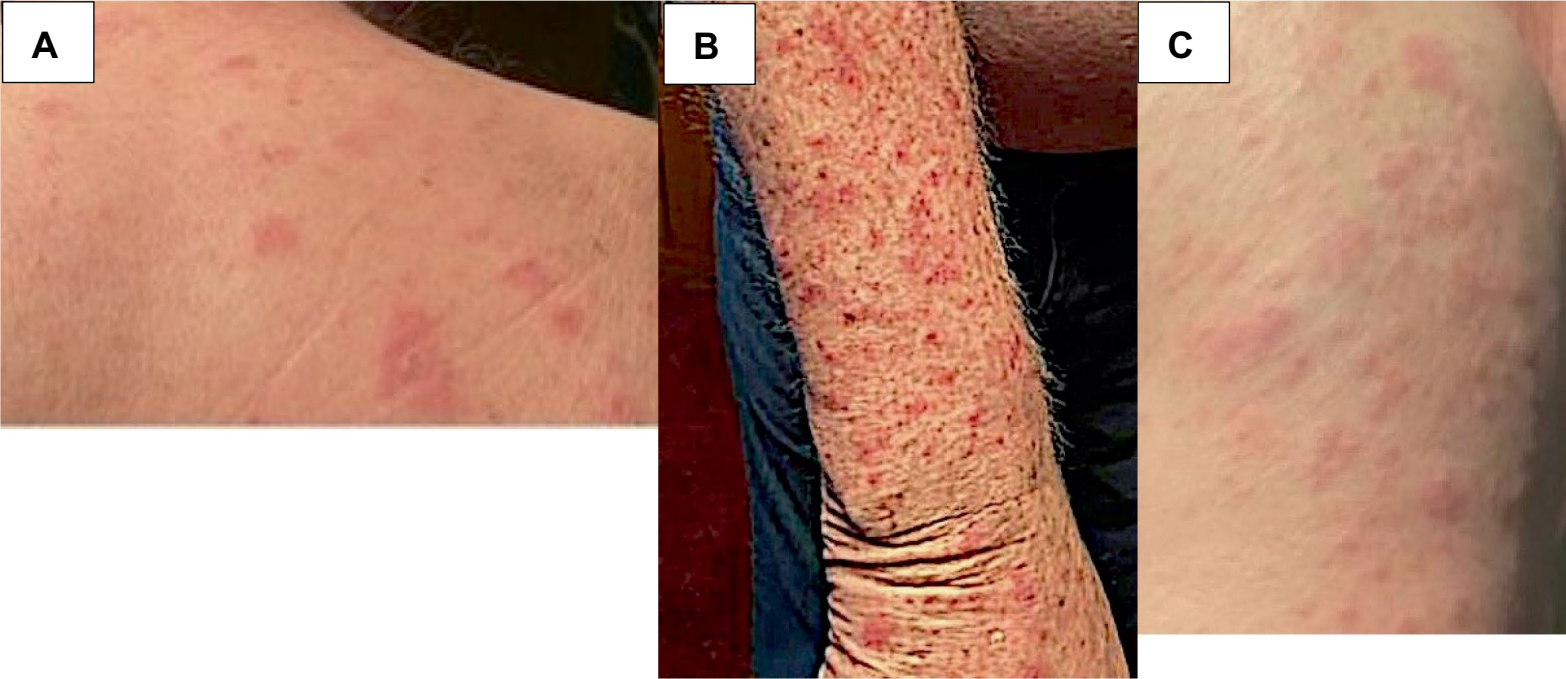
ICI-induced SCLE clinical appearance. Several photo-distributed oval pink to violaceous scaly papules and plaques on the upper back **(A)**, dorsal forearm **(B)**, and upper arm **(C)** are shown above.

**Figure 2:**
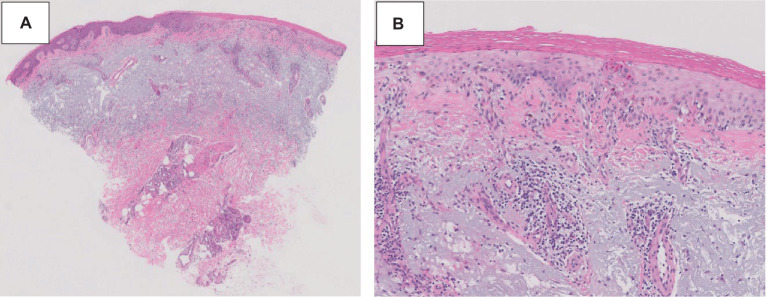
ICI-induced SCLE histopathologic appearance. Histopathologic sections of a hematoxylin and eosin-stained skin biopsy revealed vacuolar interface change along the dermal-epidermal junction associated with dyskeratotic keratinocytes and a perivascular lymphocytic infiltrate at 20× **(A)** and 100× **(B)** magnification.

A 75-year-old gentleman with no documented history of autoimmune disease presented to our clinic following onset of new photo-distributed rash. His history was significant for stage IIB gastro-esophageal junction adenocarcinoma (cT2N0M0), which was treated with neoadjuvant chemoradiation therapy followed by surgical resection. Restaging computer tomography (CT) chest, abdomen, and pelvis scans showed no evidence of disease. The patient was started on adjuvant immunotherapy with nivolumab 480 mg every 4 weeks to minimize the risk of disease recurrence. Four weeks following the first nivolumab administration, a new photo-distributed rash on his chest and bilateral upper extremities appeared. This was treated with topical triamcinolone 0.1% cream, and nivolumab treatment was withheld. One week later, he presented to the emergency department for worsening pruritic rash. The patient’s home medications included metformin, lisinopril, apixaban, metoclopramide, and omeprazole. Physical examination revealed several photo-distributed oval pink to violaceous scaly papules and plaques on the bilateral dorsal forearms, upper arms, upper chest, and upper back (Figure 1). There was no evidence of joint swelling or mucosal ulceration. Skin biopsy showed vacuolar interface dermatitis with a superficial lichenoid and perivascular lymphocytic infiltrate, consistent with a drug eruption (Figure 2). Serum laboratory testing showed an elevated anti-nuclear antibody (ANA) at 1:160 with speckled pattern, lymphopenia, and normal complement levels. Sjögren’s syndrome-A (SS-A)/Ro, SS-B/La, and dsDNA antibodies were negative. The clinicopathological presentation was consistent with ICI-induced SCLE. The patient was treated with intravenous (IV) methylprednisolone 1 mg/kg for one dose. Following IV steroids, the rash improved, and was subsequently managed with high-potency topical fluocinonide 0.5% ointment, hydroxychloroquine (HCQ) 200 mg twice daily, and prednisone 20 mg daily (slow tapering by 5 mg every 7 days). Following a discussion with the patient regarding the risks and benefits of continuing therapy, it was mutually decided to hold off on further nivolumab treatment and he was placed on active cancer surveillance. Unfortunately, 8 months later, the patient developed intraperitoneal metastatic disease, and he was subsequently enrolled in hospice care.

## Discussion

Subacute cutaneous lupus erythematosus (SCLE) is a subtype of cutaneous LE, which can present as a manifestation of SLE or independently (13). Skin lesions in SCLE are typically (1) annular (raised pink-red borders with central clearing) or (2) papulosquamous (chronic psoriasiform or eczematous) in appearance. These lesions are highly photosensitive and may involve the neck, upper chest, shoulders, arms (“V” shaped distribution), and upper back (14). The classic histopathologic pattern in SCLE is epidermal thinning, vacuolization of the basal cell layer, scattered cytoid bodies, and a perivascular lymphocytic interface dermatitis confined to the upper dermis. Dyskeratotic keratinocytes extending into the upper spinous layers is another highly characteristic finding (15, 16).

The pathogenesis of SCLE remains unclear but is thought to be multifactorial. Certain HLA types, single nucleotide polymorphisms, and epigenetic changes have been associated with increased risk (17). Ultraviolet (UV) radiation is believed to be the key mediator of SCLE. UV radiation is responsible for keratinocyte apoptosis, which leads to an inflammatory signaling cascade via increased cytokine production by plasmacytoid dendritic cells. UV radiation may also promote increased autoantigen exposure on the cell surface resulting in the formation of immune complexes, which leads to antibody dependent cell-mediated cytotoxicity. Evidence for this is supported by the frequent presence of autoantibodies in SCLE, as most patients are ANA (70%) and SS-A/Ro antibody positive (75%) (18, 19).

Numerous medications have been associated with SCLE development, including proton pump inhibitors (PPI), thiazide diuretics, angiotensin-converting enzyme inhibitors, calcium channel blockers, antifungals, and chemotherapeutic drugs (20–22). Drug-induced SCLE (DI-SCLE) lesions are indistinguishable in appearance from non-drug related SCLE (23). Patients with DI-SCLE can exhibit arthralgia or other systemic manifestations, although this is infrequent (24). More recently, ICI therapies have also been linked to SCLE (8–10). Several case reports have noted the development of SCLE in cancer patients treated with nivolumab (Table 1). For instance, Liu et al. reported a 54-year-old patient with non-small cell carcinoma (NSCLC) who developed SCLE 5 months following start of ICI treatment (25). Zitouni et al. described two cases, one patient with melanoma and the other with NSCLC who developed SCLE 2 months, and 1 month, respectively, post-ICI. Interestingly, in one of these cases, SCLE occurred 2 months following discontinuation of ICI treatment (10). Finally, in a large retrospective study of 4,487 cancer patients, there were eight cases of ICI-induced SCLE, two of which were attributed to nivolumab (26). SCLE has also been described in the literature following exposure to (1) PD-1 inhibitor, pembrolizumab; (2) PD-L1 inhibitors, atezolizumab, and durvalumab, and (3) CTLA-4 inhibitor, ipilimumab, in combination with nivolumab (8, 9, 29–35). The clinical and histopathologic features of SCLE described in these case reports resemble those observed following nivolumab administration.

**Table 1:** Management of nivolumab-induced SCLE cases in the literature.

Age/sex	Cancer	ICI to SCLE onset (cycles)	Positive Autoantibody serologies	SCLE treatment	SCLE response	ICI management	Tumor response
58 y f (25)	NSCLC	5 mo (NR)	Anti-Ro/SSA	Systemic CS, HCQ	Improvement	Rechallenge	NR
72 y f (10)	Melanoma	2 mo (post cycle 13)	ANA	Topical CS, HCQ	Improvement	Discontinuation	CR
			Anti-Ro/SSA				
			Anti-La/SSB				
43 y m (10)	NSCLC	1 mo (2)	ANA	Topical and systemic CS, HCQ	Improvement	Discontinuation	PD
			Anti-Ro/SSA				
75 y m (7)	NSCLC	2.5 mo (5)	ANA	Systemic CS	Improvement	Discontinuation	PD
			Anti-Ro/SSA				
66 y f (7)	NSCLC	12 mo (9)	ANA	Systemic CS	Resolution	Rechallenge	PD
			Anti-Ro/SSA				
60 y m (27)	SCLC	1 mo (2)	Anti-Ro/SSA	Topical and systemic CS, HCQ	Resolution	Rechallenge	PD
54 y f (28)	SCLC	20 mo (NR)	ANA	Topical CS, HCQ	Resolution	Continuation	NR
			Anti-Ro/SSA				
			Anti-La/SSB				
60 y m (28)	Melanoma	0.5 mo (NR)	ANA	Topical CS	Improvement	Continuation	NR
			Anti-Ro/SSA				

SCLE, subacute cutaneous lupus erythematosus; ICI, immune checkpoint inhibitor; CS, corticosteroids; NSCLC, non-small cell lung carcinoma; SCLC, small cell lung carcinoma; m, male; f, female; mo, months; y, year; ANA, anti-nuclear antigen; SSA, Sjögren’s syndrome-A; SSB, Sjögren’s syndrome-B; HCQ, hydroxychloroquine; NR, not reported; CR, complete response; PD, disease progression.

It remains unclear whether ICI-induced SCLE represents *de novo* cutaneous toxicity or unmasking of prior disease (36). A “multi-hit” hypothesis has been proposed to explain this phenomenon. ICI exposure may provide an additional “hit” which precipitates an augmented immune response to a previously tolerated medication (26). In a case series by Bui et al., five patients were noted to have SCLE while being treated with PD-1 or PD-L1 inhibitors. All patients were taking omeprazole, however, did not develop SCLE until they were treated with an ICI (28). In the present case, our patient was taking a PPI prior to starting nivolumab, which likely led to unmasking of his SCLE. This phenomenon has also been observed in cases of PPI-associated acute interstitial nephritis, in which ICI exposure reactivates previously quiescent self-reactive T cells (37). Additionally, tissue injury can promote epitope spreading, resulting in changes to antigen specificity, and further contributing to autoimmunity (38). Interestingly, our patient’s rash was found in the same areas where he had a history of skin cancer removal (high UV exposure and tissue injury). Finally, the direct stimulation of B-cell mediated humoral immunity by ICIs can lead to the development of autoantibodies against cutaneous antigenic targets (37, 38). Our patient had an ANA titer of 1:160, however its significance was unclear given his older age. Additionally, his autoantibody serologies were negative which is atypical for ICI-induced SCLE, as anti-Ro/SSA has been reported to be positive in up to 80%, and anti-La/SSB in 25% of cases (29).

**Figure 3:**
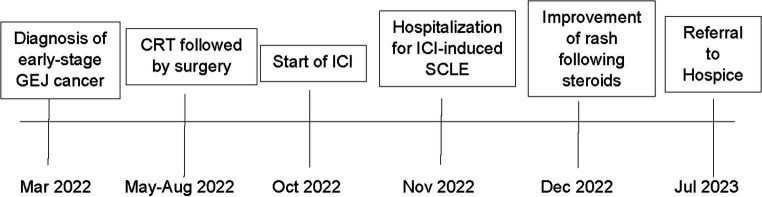
Timeline of patient’s cancer and irAE treatment course. A timeline of events for the patient is summarized above.

Given ICI exposure can cause a wide variety of skin conditions, it is crucial for clinicians to maintain a broad differential diagnosis. The most common types of cutaneous irAEs observed are: (1) nonspecific morbilliform rash, (2) pruritus, (3) papulosquamous, (4) eczematous, and (5) lichenoid reactions (39) (Figure 3). Bullous eruptions are also well described (40). Severe cutaneous adverse reactions such as Stevens-Johnson syndrome and toxic epidermal necrolysis are less common but important to recognize given the potential for significant morbidity and mortality (39). Additionally, paraneoplastic-induced SCLE (PNSCLE), which is a distinct entity from ICI-induced SCLE, should be considered within the differential diagnosis. PNSCLE has been associated with a wide variety of malignancies, frequently described in patients with lung cancer, though cases of esophageal and gastric cancers have also been reported (41). The pathogenesis of PNSCLE is not completely understood. It has been hypothesized the presence of tumor antigens, homologous to those present in the body, stimulate autoantibody production leading to an autoimmune skin reaction. For a diagnosis of PNSCLE to be suspected, it should satisfy McLean’s criteria, in which: (1) the dermatosis should develop after the malignancy, but may be present before the cancer diagnosis, (2) the dermatosis and malignancy should follow a parallel course, and (3) the rash should regress with treatment of the malignancy (42). In a published review of 37 PNSCLE cases, most patients had locally advanced or metastatic cancer. Additionally, in most patients, the SCLE lesions preceded diagnosis of their malignancy, and the lesions improved following cancer treatment (41). In the present case, a diagnosis of ICI-induced SCLE was suspected due to the clinicopathologic features of the case, the rash’s onset following ICI administration, and its improvement following treatment.

To date, there are no prospective trials which have outlined the optimal management of ICI-induced SCLE. Management of ICI-induced SCLE is dependent on several factors, including rash severity, treatment response, and the ICI therapy setting (26). Rash severity is graded according to the Common Terminology Criteria For Adverse Events (43). Patients with grade 1–2 toxicity (<30% body surface area (BSA), with or without mild symptoms), are treated with moderate-high potency topical corticosteroids. Systemic corticosteroids (prednisone 0.5–1 mg/kg daily, tapered over 4 weeks) may also be necessary. Patients with grade 3 toxicity (>30% BSA, moderate–severe symptoms), require withholding of ICI treatment, dermatology consultation, and systemic corticosteroids (prednisone 1 mg/kg daily, tapered over at least 4 weeks). Grade 4 toxicity (requiring urgent intervention or hospitalization) should be treated with IV methylprednisolone 1–2 mg/kg daily with slow tapering once the toxicity resolves and necessitates reconsideration of ICI rechallenge (40, 44). Previously reported cases in the literature have described successful management of ICI-induced SCLE using a combination of HCQ, topical, and oral corticosteroids (Table 1). Additionally, most cases did not require ICI treatment discontinuation (26). Notably, in some patients, rechallenge of ICI led to additional toxicities. For instance, Marano et al. described a patient with SCLC who developed dermatomyositis upon ICI rechallenge (44). Diago et al. described a patient with NSCLC who developed hepatitis and pneumonitis following ICI rechallenge (7). This is important, as clinicians must navigate the balance between treatment of a patient’s malignancy, and the toxicities inherent with ICI treatment (24). In the present case, the patient’s grade 3 toxicity improved following treatment with HCQ, topical, and oral corticosteroids, however after a risk–benefit discussion, it was mutually decided to hold further ICI treatment. Unfortunately, the patient experienced disease progression and was enrolled in hospice 8 months later.

## Conclusion

This paper describes a case of a 75-year-old man with gastro-esophageal adenocarcinoma and the development of ICI-induced SCLE. Drug induced SCLE is a commonly known syndrome that improves when the offending drug is stopped. SCLE secondary to ICI follows a different pattern than drug induced SCLE which enlightens additional questions about how and when to rechallenge ICI; as well as the concomitant use of drugs like HCQ and other immunosuppressants with ICI. As the use of ICIs in cancer treatment becomes more prevalent, early identification and management of ICI-induced SCLE, is essential to minimize cancer treatment interruption, and improve outcomes for these patients.

## Data availability statement

The original contributions presented in the study are included in the article/supplementary material, further inquiries can be directed to the corresponding author.

## Ethics statement

Written informed consent was obtained from the patient for the publication of this case report.

## Author contributions

AdK: Writing – original draft, Writing – review & editing. AbK: Writing – review & editing. AsM: Writing – review & editing. AlM: Writing – review & editing.
